# Environmental conditions dictate differential evolution of vancomycin resistance in *Staphylococcus aureus*

**DOI:** 10.1038/s42003-021-02339-z

**Published:** 2021-06-25

**Authors:** Henrique Machado, Yara Seif, George Sakoulas, Connor A. Olson, Ying Hefner, Amitesh Anand, Ying Z. Jones, Richard Szubin, Bernhard O. Palsson, Victor Nizet, Adam M. Feist

**Affiliations:** 1 Department of Bioengineering, University of California San Diego, La Jolla, CA USA; 2 Collaborative to Halt Antibiotic-Resistant Microbes, Department of Pediatrics, University of California San Diego, La Jolla, CA USA; 3 Department of Cellular and Molecular Medicine, University of California San Diego, La Jolla, CA USA; 4 Department of Pediatrics, University of California San Diego, La Jolla, CA USA; 5 Novo Nordisk Foundation Center for Biosustainability, Technical University of Denmark, Lyngby, Denmark; 6 Present Address: Merck & Co., Inc., South San Francisco, CA USA

**Keywords:** Experimental evolution, Bacterial evolution, Antimicrobial resistance

## Abstract

While microbiological resistance to vancomycin in *Staphylococcus aureus* is rare, clinical vancomycin treatment failures are common, and methicillin-resistant *S. aureus* (MRSA) strains isolated from patients after prolonged vancomycin treatment failure remain susceptible. Adaptive laboratory evolution was utilized to uncover mutational mechanisms associated with MRSA vancomycin resistance in a physiological medium as well as a bacteriological medium used in clinical susceptibility testing. Sequencing of resistant clones revealed shared and media-specific mutational outcomes, with an overlap in cell wall regulons (*walKRyycHI, vraSRT*). Evolved strains displayed similar properties to resistant clinical isolates in their genetic and phenotypic traits. Importantly, resistant phenotypes that developed in physiological media did not translate into resistance in bacteriological media. Further, a bacteriological media-specific mechanism for vancomycin resistance associated with a mutated *mprF* was confirmed. This study bridges the gap between the understanding of clinical and microbiological vancomycin resistance in *S. aureus* and expands the number of allelic variants (18 ± 4 mutations for the top 5 mutated genes) that result in vancomycin resistance phenotypes.

## Abstract (web summary)

Henrique Machado et al. describe mutational mechanisms associated with MRSA vancomycin resistance in *Staphylococcus aureus* using adaptive laboratory evolution experiments focused on tolerance. Their results reveal environment-dependent mutational strategies to vancomycin tolerization and the impact of mutations in regulatory genes, providing insight into the development of antibiotic resistance under multiple conditions.

## Introduction

Antibiotic resistance is a global healthcare threat worldwide^1,2^. Consequently, numerous strategies have been developed and implemented to monitor, assess, and circumvent the development of antibiotic resistance among pathogens^3–5^. Continual monitoring and assessment are key to getting a global picture of the problem and for increasing our understanding of the mutational mechanisms that pathogens employ toward resistance development.

Although somewhat successful, current monitoring and assessment approaches are based on existing pathogen-specific knowledge. Because mechanisms for antibiotic resistance evolution are poorly defined, full realization of critical threats often occurs only after resistance has emerged. Importantly, currently known allelic variations associated with reduced susceptibility to a given antibiotic are not comprehensive. This means that additional variations of a given allele or variation in other alleles can result in the same phenotype^6^. Furthermore, bacterial susceptibility to antibiotics is measured following guidelines of the Clinical & Laboratory Standards Institute (CLSI), which recommends using the bacteriological rich media cation-adjusted Mueller–Hinton broth (CA-MHB) to determine antibiotic susceptibility. CA-MHB was specifically developed for its ability to reliably support the cultivation of common human pathogens from clinical samples, and only later adopted for minimum inhibitory/bactericidal concentration (MIC/MBC) testing of antibiotic candidates. However, CA-MHB does not come close to recapitulating the environment encountered by bacteria in vivo and has been shown to be less reliable in predicting in vivo activity of antibiotics than other more physiological media such as mammalian tissue culture media^7–9^. Adaptive laboratory evolution (ALE) is a strategy that allows the investigator to address both issues of limited coverage of allelic variation and environment-specific susceptibility through the study of identification of causal mutational mechanisms^10–12^. ALE leverages microbial growth under different environments and conditions, wherein the natural mutation rate of bacteria can be exploited to sample successful allelic variations.

*S. aureus* is an historical example of the successful development of antibiotic resistance by a common human pathogen, with methicillin-resistant strains (MRSA) presenting significant treatment challenges^13–15^. The most commonly recommended drug for the treatment of MRSA infections is the glycopeptide vancomycin^16,17^. Even though very few vancomycin-resistant MRSA clinical isolates have been reported^18–20^, an increasing challenge of clinical treatment failures is well documented^15^. The established MIC breakpoints determined by CLSI classify *S. aureus* into three susceptibility categories: vancomycin-susceptible *S. aureus* (VSSA, MIC ≤ 2 μg/mL), vancomycin intermediate-resistant *S. aureus* (VISA, MIC = 4–8 μg/mL), and vancomycin-resistant *S. aureus* (VRSA, MIC ≥ 16 μg/mL).

Here we used a clinical MRSA isolate (TCH1516), isolated from an adolescent with severe sepsis^21^, and applied adaptive laboratory evolution (ALE) to uncover mutational mechanisms associated with resistance under two different environmental conditions: (i) CA-MHB, the nutrient-rich bacteriological medium used for clinical susceptibility testing by CLSI recommendations; and (ii) Roswell Park Memorial Institute medium (RPMI), a mammalian cell culture medium that better mimics human physiology^22^. We further phenotypically characterized several vancomycin-tolerant and -resistant clones and identified genetic mutations associated with such adaptations. The mutational evolutionary pathways toward vancomycin tolerance exhibited media specificity, with an overlap in regulatory rearrangements in cell wall regulons. We also establish that vancomycin-resistant phenotypes that developed in physiological media do not translate into resistance in bacteriological media, where a major resistance mechanism relies on change of the cell surface charge, possibly associated with mutation of *mprF*. This study significantly expands knowledge of allelic variation that contributes to *S. aureus* vancomycin tolerance.

## Results

### Tolerization of *S. aureus* TCH1516 to vancomycin

**Fig. 1:**
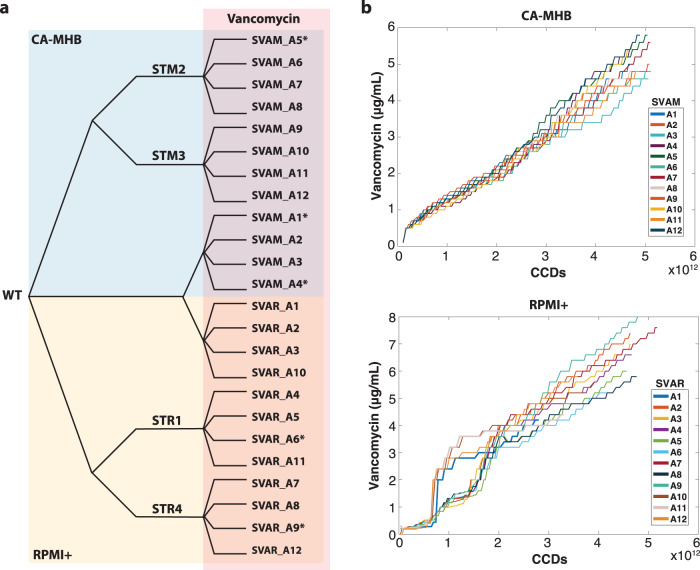
Tolerization adaptive laboratory evolution. **a** Experimental design. Wild-type (WT) *S. aureus* TCH1516 was used as starting strain along with strains which were initially adapted to each media condition^12^. Strains were tolerized under CA-MHB (blue) and RPMI+ (yellow) media conditions with increasing concentrations of vancomycin (red). Isolate naming from each lineage is listed. **b** Plots showing the stepwise increase of vancomycin throughout the TALE experiments. * denotes hyper-mutators. STM: *Staphylococcus aureus* adapted to CA-MHB. STR: *Staphylococcus aureus* adapted to RPMI+. SVAM: *Staphylococcus aureus* tolerized to vancomycin in CA-MHB. SVAR: *Staphylococcus aureus* tolerized to vancomycin in RPMI+. CCDs: cumulative cell divisions.

Adaptive laboratory evolution (ALE) relies on the natural capability of cells to adapt to new environments. Here, we have applied this technology to engender tolerance of *S. aureus* TCH1516 to vancomycin, and unravel the molecular mechanisms for this adaptation, in a so-called Tolerization ALE (TALE)^10,12^. Two media types were used in this experiment: CA-MHB, an undefined nutrient-rich bacteriological medium used for clinical susceptibility testing and the well-defined Roswell Park Memorial Institute medium (RPMI), routinely used in the culturing of mammalian cells, and which resembles the physiological conditions in the human body^22^, supplemented with 10% bacteriological rich Luria-Bertani medium (RPMI+) to ensure robust bacterial growth. For each media type, four replicates of wild type (WT) and of two media-adapted clones were evolved to stepwise increasing concentrations of vancomycin (Fig. 1a). Media-adapted clones were previously developed^12^ and were used as a baseline to understand media-specific adaptations versus antibiotic resistance development. The TALE experiments were conducted for ~30 days and ~5 × 10^12^ cumulative cell divisions (CCDs). The final vancomycin concentrations reached an average of 5.14 ± 0.46 μg/mL in CA-MHB and 6.13 ± 1.03 μg/mL in RPMI+ (Fig. 1b), compared to a tenth of the MIC used as start concentration (MIC in Supplementary Table 1; starting concentration of 0.1 and 0.2 μg/mL, in CA-MHB and RPMI + TALEs, respectively).

### Phenotypes and tradeoffs in vancomycin tolerization

**Fig. 2:**
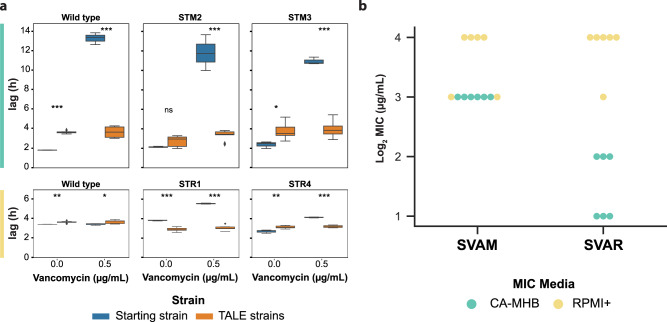
Phenotypic changes observed in vancomycin-tolerized strains. **a** Box plots displaying growth tradeoffs grouped by the starting strain for each set of TALE experiments. Evolution starting strains and vancomycin adapted strains were grown in the absence and presence of vancomycin. Assessment of the lag-phase duration of starting strains and evolved strains in the same media as that used for tolerization, CA-MHB (top, green) and RPMI+ (bottom, yellow). Error bars represent one standard deviation. Evolved strain averages used 2, 3, or 4 distinct clones derived from the given starting strain (Supplementary Table 1), all determinations were made in triplicate. Values that are significantly different by ANOVA are indicated by asterisks (ns, non-significant; *P ≤ 0.05; **P ≤ 0.01; and ***P ≤ 0.001) (Supplementary Table 3). An example of one of these growth curves, comparing pre-evolved and vancomycin adapted strain is presented in Supplementary Fig. 2. **b** A plot of Log_2_ vancomycin MIC values of evolved strains from all TALE conditions tested in both media types. SVAR strain tolerance phenotypes in RPMI+ were not maintained when tested in CA-MHB media. SVAM: *S. aureus* tolerized to vancomycin in CA-MHB. SVAR: *S. aureus* tolerized to vancomycin in RPMI+.

The TALE evolved strains, adapted for growth in increasing concentrations of vancomycin, were evaluated for changes to their growth phenotypes and antibiotic susceptibility. Growth rate was not affected in TALE strains, but an increase in the lag phase could be identified in CA-MHB-evolved strains when measured without vancomycin stress and compared to their pre-evolved counterparts (Fig. 2a). As expected, TALE strains grew in higher concentrations of vancomycin, with an increase in MIC of up to 8-fold (Supplementary Table 1). Previous studies have outlined the phenotypic characteristics of clinically isolated vancomycin-tolerant strains^15,23^. We observed similar characteristics in the TALE-evolved strains, including lower hemolytic activity, reduced autolysis, and increased cell-wall thickness (Supplementary Fig. 1 and Supplementary Table 2). Further, vancomycin-tolerant strains generated in the laboratory have been reported to be phenotypically unstable^24^, losing their tolerance after growth in non-selective conditions. Therefore, we grew the vancomycin-tolerized strains for 21.79 ± 2.08 passages (9.41 × 10^11^ ± 9.84 × 10^10^ CCDs) in the media used for evolution, without vancomycin. The endpoint strains were generally stable in maintaining their tolerance phenotype in 11 out of 12 lineages, with the exception of the SVAM_A10 lineage, which decreased its MIC from 8 to 2 μg/mL (Supplementary Table 1).

TALE strains lost vancomycin susceptibility, with a corresponding increase in their MIC (Supplementary Table 1). We further assessed if this decrease in susceptibility held true across the different media environments. Although CA-MHB vancomycin-tolerized strains (i.e., SVAM) maintained their tolerance phenotypes in RPMI+, the same was not true for the clones evolved in RPMII+. RPMI+ vancomycin-tolerized strains (i.e., SVAR) did not show decreased susceptibility when screened in CA-MHB media (Fig. 2b). Thus, the strains evolved under RPMI+ displayed a media-specific tolerance phenotype, as compared to the translatable phenotype of the CA-MHB derived strains, for the two media conditions tested here.

### Environment dependent mutational strategies to vancomycin tolerization

For each of the 24 independent adaptive evolutionary lineages, 2–3 clones were randomly selected at different time points of the TALE experiments and were sequenced for mutational analysis. For each media type, ~400 unique mutations could be identified (457 in CA-MHB and 374 in RPMI+, *n* = 50) (Supplementary Data 1 and 2), with the majority (~85%) being single nucleotide polymorphisms (SNPs). The percentage of transitions and transversions was quite similar (55% transitions, 45% transversions), but there was a bias in mutations from GC to AT, which constituted about 40% of the SNPs compared to 20% from AT to GC. The observed AT-biased mutation has been shown to be universal for bacteria, independent of their genomic GC content^25–27^.

**Fig. 3:**
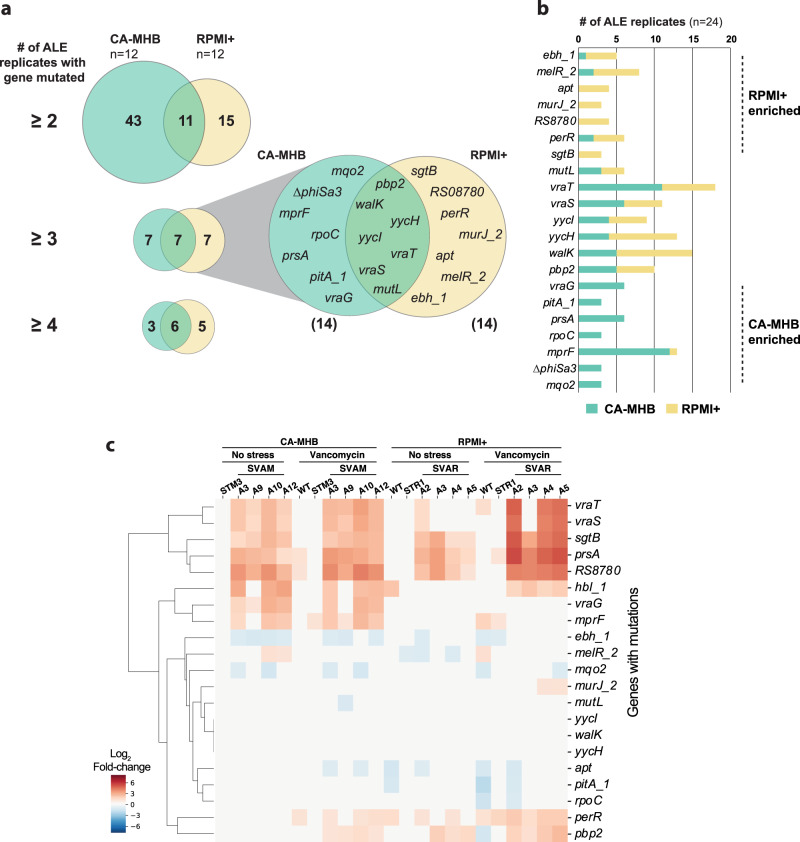
Key mutations found in MRSA during vancomycin tolerization. **a** Venn diagrams of the number of key mutated genes in the two utilized media conditions (i.e., CA-MHB and RPMI+). **b** A bar plot of the number of lineages with mutations in a key mutated gene, *n* = 24 lineages, 12 for each media condition. **c** A heatmap of RNAseq expression level of key mutated genes in a selection of starting and TALE-derived strains, in the presence and absence of vancomycin. STM: *Staphylococcus aureus* adapted to CA-MHB. STR: *Staphylococcus aureus* adapted to RPMI+. SVAM: *Staphylococcus aureus* tolerized to vancomycin in CA-MHB. SVAR: *Staphylococcus aureus* tolerized to vancomycin in RPMI+.

Key mutated genes were considered to be those mutated in two or more independent TALE lineages and that were present in at least one clonal sample. If a gene was mutated in multiple flasks of the same TALE lineage or was only observed in sequenced population samples, it was not considered. A total of 69 key mutations were identified for both media types (Supplementary Fig. 3). Overall, clones evolved in RPMI+ typically had a lower number of mutations (7.4 ± 3.4 mutations per strain) compared to those evolved in CA-MHB (10.6 ± 4.6 mutations per strain), excluding hyper-mutators. This mutational count difference is also reflected in the number of key mutated genes identified in both conditions, 54 versus 26 in CA-MHB and RPMI+, respectively (Fig. 3a). By increasing the threshold of lineages with a given gene mutated, the decrease in the number of key mutated genes in CA-MHB was striking, whereas in RPMI+ there was less variance (Fig. 3a). Although the *apt* gene appears as a key mutation in RPMI+ because it was mutated in all four replicates that were started from wild type, this mutation has been associated with a growth rate increase in RPMI+ and does not have an effect on antibiotic susceptibility^12^. Furthermore, mutations in *mutL* (encoding a DNA mismatch repair protein) were found in TALE strains from both media conditions, and these strains displayed hypermutator phenotypes similar to previous reports^28,29^. The hypermutator strains had a higher number of mutations, compared to other TALE strains. However, the mutations identified were not distinctly linked with vancomycin tolerance, but rather randomly spread throughout the genome (Supplementary Data 1 and 2).

Distinct key mutations were identified in both bacteriological and physiological media conditions, with the overlap of similar mutations being mostly in regulatory genes, specifically in the *vra* and *wal* regulatory systems (Fig. 3a). Mutations in these two systems have been previously associated with decreased susceptibility to glycopeptides^15,24,30,31^. For the *vra* system, composed of the genes *vraSRT*, we identified 37 different mutations, of which 5 exact mutations (VraT-A151T, VraS-A314V, VraS-G88D, VraS-T264A, and VraR-V14I) and 3 mutated positions (VraT-P126, VraT-N74, and VraS-V66) have been previously described^30–33^. For the *wal* system, composed of the genes *walKRyycHI*, we identified 44 mutations, 26 of which were in the accessory genes *yycHI* (21 resulting in possible pseudogenization, i.e., gene disruption). This pseudogenization type of gene disruption (specifically, a frameshift mutation resulting in truncation) had been previously observed in an in vivo evolution study in a patient^13^. From the 18 mutations in *walKR*, three mutated positions have been previously described (WalK-G223, WalK-S221, WalK-V383), which in previous studies resulted in different amino acid substitutions at these positions^33–37^. The mutated alleles strongly correlate with those found in clinical isolates, although most of the specific mutations are different, contributing to an expansion of the tolerance allelome.

Vancomycin targets the cell wall, therefore, the finding that most of the key mutated genes under both TALE media conditions were related to cell wall biosynthesis was expected (i.e., *sgtB, prsA, walKRyycHI, vraSRT, pbp2, murJ_2, mprF*)^38–45^. For example, PBP2 is the only bifunctional *S. aureus* penicillin-binding protein (transglycosylase and transpeptidase activities)^46,47^ and is involved in cell wall cross-linking. PBP2 has also been associated with susceptibility to membrane and cell-wall targeting antibiotics^43,48^. Besides the shared mutations, there were a number of media-specific mutations (Fig. 3b). This environmental dependency was also evident from the expression of these key mutated genes (Fig. 3c). For example, *vraG* and *mprF* genes that are mostly mutated in CA-MHB condition, were highly expressed in vancomycin-tolerized strains in the same media, while there was no differential expression in RPMI+. On the other hand, some genes that seem to be mutated in a condition-specific manner presented a similar transcriptional profile in both (e.g*., sgtB* and *prsA*). Again, most of the key mutated genes were related to cell wall biosynthesis (e.g., *sgtB, prsA, walK, vraT, pbp2, mprF*). However, there were other key mutated genes associated with transcription (*rpoC*), transport (*pitA_1, vraG*), regulation (*perR, melR_2*), metabolism (*mqo2*), pathogenesis (*ebh_1*), and unknown function (*RS08780*).

**Fig. 4:**
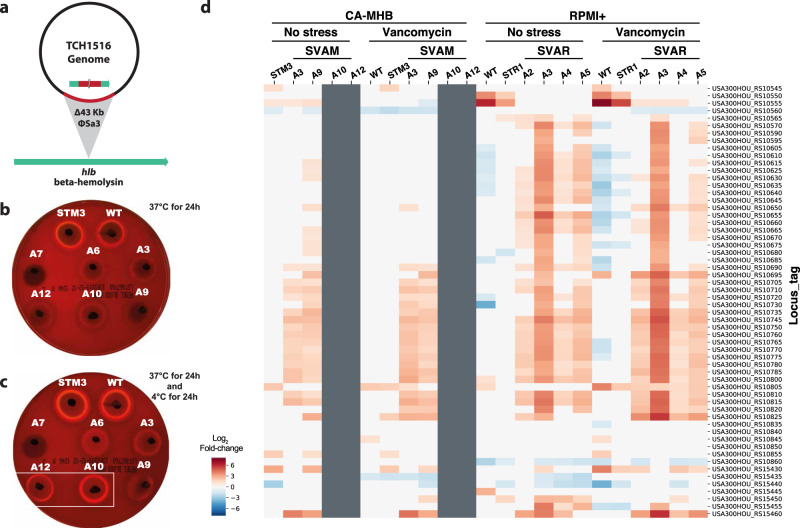
The excision of ΦSa3 prophage. **a** Schematic representation of the excision of the ΦSa3 prophage from the TCH1516 genome which leads to the repair of *hlb* gene, encoding for a β-hemolysin. **b** An image of a plate displaying hemolytic activity after 24 h incubation at 37 °C for vancomycin TALE strains in CA-MHB and their corresponding starting strains, wild-type (WT) TCH1516 and CA-MHB media-adapted STM3. **c** An image of the same plate in (**b**) displaying hemolytic activity following an additional 24 h incubation at 4 °C, to assess cold hemolytic activity of β-hemolysin. Increased hemolytic activity can be seen for strains SVAM_A10 and SVAM_A12 (boxed). **d** A heatmap of RNAseq expression levels of genes encoded within the prophage ΦSa3. Gray indicates absence of the gene in a strain due to excision of the prophage. STM: *Staphylococcus aureus* adapted to CA-MHB. STR: *Staphylococcus aureus* adapted to RPMI+. SVAM: *Staphylococcus aureus* tolerized to vancomycin in CA-MHB. SVAR: *Staphylococcus aureus* tolerized to vancomycin in RPMI+.

Mutational analysis of TALE clones from CA-MHB revealed an instance of parallel evolution involving excision of the prophage ΦSa3 in three independent lineages. Large identical genomic deletions of 43,048 bp resulted from the excision of prophage ΦSa3, the most prevalent prophage family in *S. aureus*^49^, which encodes for the immune evasion cluster^50,51^. This cluster harbors the immune modulators staphylokinase (Sak), staphylococcal complement inhibitor (SCIN), staphylococcal enterotoxin A (Sea), and chemotaxis inhibitory protein of *S. aureus* (CHIPS)^51–53^. Excision of the prophage results in the repair of the β-hemolysin gene (*hlb*) (Fig. 4a)^54^. Selective excision of prophage ΦSa3 has been reported, suggesting it acts as a molecular regulatory switch for β-hemolysin production^54^. Testing of hemolytic activity of two of the three TALE strains and their starting strain counterparts confirmed one other characteristic phenotype observed in VISA strains, reduced hemolytic activity (Fig. 4b). Since *hlb* encodes for a cold-active hemolysin, after 24 h incubation at 4 °C, it was possible to observe acquired hemolytic activity for the TALE strains that have excised the prophage ΦSa3 (Fig. 4c), strains SVAM_A10 and SVAM_A12. In contrast, none of the RPMI+ media tolerized strains excised the prophage (Fig. 3b). The expression of genes encoded in the prophage was analyzed in TALE strains derived from both media environments and it was observed that there was higher transcriptional activity of prophage genes in RPMI+ as compared to CA-MHB, for strains which retained the prophage genes (Fig. 4d). This observation suggests an advantage in maintaining these prophage genes in RPMI+ upon vancomycin stress.

### Broad-scale impact of mutations in regulatory genes

**Fig. 5:**
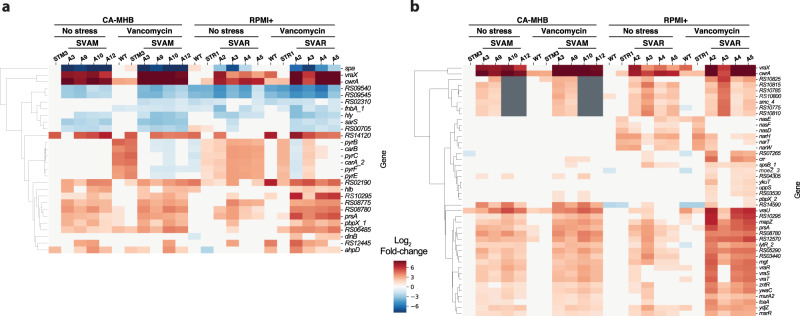
Rearranged transcriptional landscapes of operons associated with vancomycin tolerance in TALE strains. **a** A heatmap of expression levels of the genes in the WalR regulon^57^ displaying significant levels of differential expression, both up and down. **b** Expression of genes in the VraR cell wall stimulon^55^. STM: *Staphylococcus aureus* adapted to CA-MHB. STR: *Staphylococcus aureus* adapted to RPMI+. SVAM: *Staphylococcus aureus* tolerized to vancomycin in CA-MHB. SVAR: *Staphylococcus aureus* tolerized to vancomycin in RPMI+.

The most commonly mutated genes in both media conditions during the TALE experiments were annotated with regulatory functions, with the *walRKyycHI* and *vraRST* operons being the most targeted (Fig. 3b). Interestingly, the accessory genes (i.e., *yycH*, *yycI*, and *vraT*) known to impact the activity of these regulators^55,56^ were some of the most often mutated. Mutations in regulatory genes tend to impact bacterial responses on a broad scale, which is difficult to assess solely from mutational data. Therefore, we performed RNAseq with and without vancomycin stress to understand how the mutations observed impacted the transcriptional profile of the evolved strains. Within the previously characterized WalR regulon^57^, there were several genes differentially regulated in the vancomycin TALE clones. In fact, both upregulation and downregulation were observed in several genes within this regulon (Fig. 5a). Downregulation of the *spa* gene and lower hemolytic activity are a characteristic of VISA strains^15^, which was also confirmed from the acquired transcriptional data (Fig. 5a). The pyrimidine operon has been deemed important for growth in RPMI+^58^ and was upregulated in strains evolved in this medium. The VraR regulon, responsible for the control of the cell wall stimulon, showed an overall upregulation in vancomycin-adapted strains (Fig. 5b). The most upregulated genes, *vraX* and *cwrA*, are described to be part of both regulons (Fig. 5), and have both been linked to cell wall stress response^59,60^. Even though all the characterized vancomycin-tolerized strains presented a similar transcriptional rearrangement of these regulons, the analyzed strains carried distinct mutations in these regulatory genes (35 and 42 total different mutations in the *vra* and *wal* regulons, respectively; Supplementary Data 1 and 2), suggesting that multiple mutational mechanisms can result in a similar transcriptional landscape. Overall, the TALE-derived mutations in these regulatory systems form a defined set of multiple unique mutations and resulted in significant rearrangements in expression levels of genes strongly associated with the observed tolerance phenotypes.

### Elucidation of molecular mechanisms in vancomycin tolerance

**Fig. 6:**
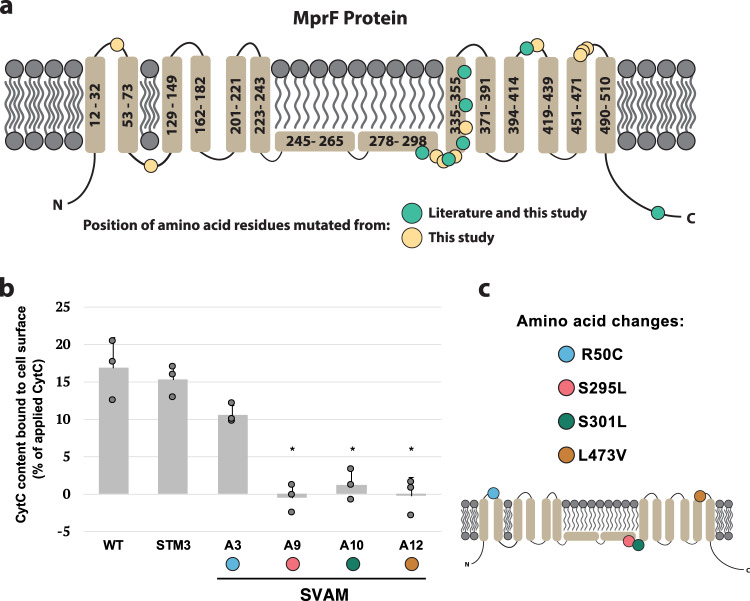
MprF mutations and their effect on cell surface charge. **a** Topology of the MprF membrane protein and mapping of mutations identified in this dataset (yellow and green circles), in comparison to those previously reported (green circles)^62^. **b** A graph quantifying the cell surface charge of strains tolerized to vancomycin, in comparison to their starting strain counterparts, *n* = 3 biologically independent samples. Values that are significantly different (*P* ≤ 0.05) from the value for the respective starting strain (WT or STM3) by Student’s *t*-test are indicated by an asterisk. Error bars represent one standard deviation. **c** Identified locations of the specific amino acid substitutions observed in the tolerized strains and their position in the MprF structure. These mutations correspond to the strains tested in panel (**b**). STM: *Staphylococcus aureus* adapted to CA-MHB. SVAM: *Staphylococcus aureus* tolerized to vancomycin in CA-MHB.

Multiple mutations in *mprF* were observed in CA-MHB vancomycin-tolerized strains and were validated to display a significant decrease in negative cell surface charge. *mprF* encodes for a multi-peptide resistance factor and has been associated with increased resistance to antimicrobial peptides and cell-wall or membrane-acting antibiotics, such as daptomycin, oxacillin, and vancomycin^45,61,62^. Besides the previously described regulatory gene mutations, *mprF* was the most mutated gene in strains tolerized to vancomycin in CA-MHB. In the 12 CA-MHB-tolarized lineages, we identified seven previously reported mutations that led to resistance through a MprF mechanism and extended this knowledge base by identifying 10 new mutations (Fig. 6a). The MprF described resistance mechanism consists of decreasing the negative cell surface charge, and therefore repulsing cationic molecules^62^. Since vancomycin is also a positively charged peptide, one can speculate that this general mechanism would lead to increased vancomycin tolerance. In order to support this, we characterized the cell surface charge in tolerized mutants and its starting strain counterparts (Fig. 6b). Tolerized mutants, with amino acid changes toward the C-terminal part of MprF, had a significant decrease in negative cell surface charge (Fig. 6b and c). Strain SVAM_A3, with an amino acid change at position 50 (R50C), did not have a significant change in cell surface charge, which explains the observation of mutational hot-spots between positions 278 and 510 (Fig. 6a). Thus, a similar mechanism of shifting the cell surface charge from more to less negatively charged was validated for a subset of the *mprF* mutants uncovered from the TALE experimentation in this study.

### Association of genetic targets with decreased susceptibility

Gene inactivation enables the assessment of a given gene’s role in tolerance. In order to understand the importance of the mutated and highly expressed genes in vancomycin tolerance, we relied on the available *S. aureus* Nebraska Transposon Mutant Library^63^. We identified five genes required for tolerance and five genes impairing tolerance. A selection of mutants in key mutated genes or highly expressed genes was evaluated for their vancomycin susceptibility in both environmental conditions (Table 1). Interestingly, reduced vancomycin susceptibility was seen for all strains when testing was performed in RPMI+ compared to CA-MHB, in some instances with twofold or greater increases in vancomycin MIC in RPMI+. Media-specific susceptibility was observed for several mutants, similar to what was observed with the TALE-derived clones. Transposon inactivation of genes *yycI*, *sgtB*, *melR_2*, *stp*, and *lytM_2* resulted in a decreased susceptibility to vancomycin in RPMI+, but no difference in CA-MHB. On the other hand, *vraT*, *vraR*, *vraS*, *vraG*, and *mprF* showed an increased susceptibility to vancomycin in CA-MHB, but no difference in RPMI+. The fact that gene inactivation can lead to decreased susceptibility is noteworthy, as 57 (15.3%) and 76 (16.5%) of the total mutations identified lead to likely gene disruption in the RPMI+ and CA-MHB datasets, respectively. It is also noteworthy that some of the gene disruptions identified in our datasets were in the same genes that showed here a higher MIC upon inactivation, specifically *yycI* and *stp* (in both media conditions), *melR_2* (only in RPMI+), and *lytM_2* (only in CA-MHB) (Supplementary Data 1 and 2). These findings not only confirm the role of several genes (i.e., *yycI*, *sgtB*, *melR_2*, *stp*, *lytM_2, vraT*, *vraR*, *vraS*, *vraG*, and *mprF*) in vancomycin tolerance, but also highlight the different roles these have in the development of resistance under varying environmental conditions.

**Table 1:** Minimum inhibitory concentration (MIC_90_) of selected strains from the *S. aureus* Nebraska Transposon Mutant Library^63^ in both environmental conditions, CA-MHB and RPMI+.

		MIC_90_ (μg/mL)	
Strain ID	Gene	CA-MHB	RPMI+
JE2	–	1	2
NE1693	*yycH*	1	2
NE1865	*yycI*	1	4
NE596	*sgtB*	1	4
NE1304	*melR_2*	1	4
NE1919	*stp*	1	4
NE641	*lytM_2*	1	4
NE274	*vraT*	0.5	2
NE554	*vraR*	0.5	1–2
NE823	*vraS*	0.5	2
NE70	*vraG*	0.25–0.5	2
NE1360	*mprF*	0.25–0.5	2
NE665	*perR*	0.5–1	2
NE387	*cwrA*	1	2

Strain ID refers to the NE identifier and the wild-type strain used in the generation of the transposon mutants (JE2). Gene indicates the gene that has been disrupted by the transposon.

## Discussion

The expanding problem of antibiotic-resistant pathogens has been discussed for almost a century^64^, but we remain in the infancy of understanding its complexity. In this study, we sought to understand changes in susceptibility, mutational mechanisms which enable such changes, and phenotypic responses of tolerant strains by analyzing growth screens, transcriptomics, and specific assays for evolved and mutated strains of MRSA under multiple media conditions. The main findings from this work are that (i) TALE successfully generated tolerant strains in both media types, and while tolerance phenotypes translated across media types for CA-MHB TALE-derived strains, the same was not true for RPMI+; (ii) analysis of key mutational mechanisms revealed that numerous genetic allele variations can lead to similar transcriptional and phenotypic changes, especially in the CA-MHB condition; and (iii) TALE-derived strains shared similar properties to resistant clinical isolates in phenotype, mutation types, and gene expression.

There were a number of specific findings that provide context for the main conclusions of this study. These include: (i) the majority of TALE-derived strains tested maintained a tolerant phenotype after prolonged evolution under a no vancomycin stress condition; (ii) greater heterogeneity was found in the mutations observed in TALE-derived strains to become tolerant in CA-MHB than RPMI+, with a set of shared mutational mechanisms on the gene level from TALE-derived strains under both media conditions (enriched in cell wall-related regulators), which resulted in major changes in expression for the operons they regulate even though the unique alleles differed; (iii) mutations in *mprF* are a key mechanism in CA-MHB, which decreased the overall negative cell surface charge and hypothetically limited vancomycin access to the cell wall; (iv) similar to clinically isolated strains, TALE-derived strains had lower hemolytic activity and reduced autolysis^15^, mutations of the nature of pseudogenization^13^, and similar transcriptional changes for virulence-associated genes (e.g., *spa*^65,66^, *agr*^13,67^); and last, (v) different genetic targets have enhancing or impairing roles in tolerance, depending on the environmental condition. Taken together, these findings provide specific information for a change in susceptibility for an important pathogen, an antibiotic commonly used to treat it, and media conditions relevant for human physiology and antimicrobial testing. Moreover, they provide context for the overall development of antibiotic resistance under multiple conditions and independent lineages that can be used to understand the issue as a whole.

The environmental conditions analyzed here represent both the in vivo human physiological environment, and the environment under which standard antimicrobial susceptibility is performed in the clinical laboratory. We showed that the evolutionary strategies adopted in each condition overlap on two major regulatory systems, VraRS and WalRK, which are responsible for the homeostasis of the cell wall^42,55,68,69^ and had been previously linked to glycopeptide resistance^15,30,31^. More interesting is the fact that besides these regulatory mutations, which seem to confer similar transcriptional rearrangements, other mutations were largely media-dependent, suggesting that specific media restricted the evolutionary process differently. We have previously shown that growth of *S. aureus* in RPMI+ and CA-MHB led to large transcriptional landscape changes^58^. In RPMI+ the buffering system used is bicarbonate, a ubiquitous buffer found in humans, which has been shown to potentiate the activity of several antibiotics by dissipating the proton motive force in bacteria^7–9^. This process might be one of the major reasons for the distinct evolutionary strategies observed under different media. For instance, this is likely the reason why we do not observe *mprF* mutations in the RPMI+ evolved strains, but they are seen in all the CA-MHB TALE lineages. MprF activity leads to the alteration of the cell surface charge^61,62^, but in the case of RPMI+ the membrane proton motive force is already compromised by the bicarbonate buffer system^7^, making it an evolutionarily less viable solution toward vancomycin tolerance in this media. The tolerance mechanism through *mprF* mutation also explains the extended lag phase observed for all the strains tolerized in CA-MHB media, since an altered cell surface charge impacts bacterial cell division^70,71^. We must note that for other antibiotics, such as daptomycin, *mprF* mutations have been reported in the clinic and are believed to drive increased tolerance to this lipopeptide^45,61,62^; but here we show that mutations in this gene are not the most favorable evolutionary path for vancomycin tolerization in RPMI+.

Even though both media used in vancomycin tolerizations resulted in highly tolerant vancomycin strains, in the case of strains evolved in RPMI+, this phenotype did not translate to CA-MHB media. Media-dependent susceptibilities have been previously reported^7–9,72^. Here we showed that the evolution of resistance in physiological conditions is not phenotypically revealed in clinical susceptibility testing (Fig. 2b). The fact that decreased vancomycin susceptibility acquired in RPMI+ simulates the selective conditions under which it evolves in patients receiving vancomycin^22^, and that these changes are not detected in the CA-MHB utilized laboratory testing, essentially shows that the clinical laboratory is blind to the clinically relevant reduction in vancomycin susceptibility that evolves in *S. aureus*. This may explain the poor clinical efficacy of vancomycin even against *S. aureus* isolates that the laboratory designates as susceptible. Clinical experience abounds for patients with MRSA bacteremia caused by organisms fully susceptible to vancomycin, yet their infections still fail to clear despite adequate dosing. The clinical laboratory shortcomings in detecting vancomycin resistance in *S. aureus* may be one explanation for vancomycin being unique among anti-staphylococcal antibiotics in that resistance took decades to emerge (at least according to the laboratory). Resistance to every anti-staphylococcal antibiotic has emerged just a few years after the clinical introduction of that antibiotic, yet for vancomycin, which was introduced into clinical practice in 1958, VISA was not described until 1997^73^. Examining these data at an even higher level shows that every mutant from the Nebraska library as well as TCH1516 wild type demonstrated a higher vancomycin MIC in RPMI+ compared to CA-MHB. Given that the area under the curve (AUC)/MIC ratio is the pharmacokinetic target reflective of vancomycin activity suggests considerably weaker activity of vancomycin in vivo than indicated in clinical laboratory conditions^74^. Indeed, the well-described clinical-microbiological discordance of vancomycin with regards to *S. aureus* explained by our findings supports a serious re-examination of how antimicrobial susceptibility paradigms can be made more clinically relevant^8^.

During the many years that antibiotic resistance has been studied, associations have been made between specific alleles and decreased antibiotic susceptibility^13,15,23,30,32,34–36^. Numerous surveys have been conducted using PCR and sequencing to determine the likelihood of a given strain to be less susceptible to a given antibiotic^31,75–77^. Here we showed that limiting the analysis to a handful of genes can be misleading and that many allele variants can result in the same outcome. We identified several mutations in alleles previously associated with decreased susceptibility, with most of the mutations being new variants. A high-throughput approach utilizing ALE was an efficient way to sample the evolutionary pathways available for the development of antibiotic resistance, while expanding on the knowledge of allelic variation responsible for such phenotypes. Examples provided here are the mutations in the regulatory systems (i.e., *vraSRT* and *walKRyycHI*) and in *mprF*. We showed that different allele variants in regulatory genes can similarly impact the transcriptional landscape. We have largely expanded the knowledge of *mprF* allele variants that result in altered cell surface charge that might lead to decreased vancomycin susceptibility, thus bridging the knowledge gap between vancomycin and peptide antibiotic cross-resistance.

Pseudogenization is another type of genetic variance we believe merits attention. We previously showed that *S. aureus* restored pseudogenes in order to overcome metabolic limitations^78^. In this dataset, ~15% of all the mutations led to pseudogenization, which can translate into decreased susceptibility, as demonstrated with the transposon mutants (Table 1). This strengthens the hypothesis that *S. aureus* can use this pseudogenization mechanism to adapt to distinct environments, including the development of antibiotic resistance. A study looking at the adaptive evolution of *S. aureus* during chronic endobronchial infection of a cystic fibrosis patient over 26 months identified 391 mutations (comparable to our datasets) with none of the mutations predicted to result in pseudogene formation^79^. The rates at which pseudogenization occurs and reverts requires further experimental evidence in order to support this method as a common evolutionary strategy in *S. aureus*.

In conclusion, the application of ALE to develop *S. aureus* strains tolerized to vancomycin was successful and links can be drawn between TALE-derived strains and clinical isolates. Susceptibility was not only decreased for the targeted antibiotic, but it also reproduced phenotypes similar to those previously reported for clinical strains with decreased vancomycin susceptibility^15^. Furthermore, it allowed us to understand evolutionary strategies and constraints in two clinically relevant media environments while expanding our knowledge of the diversity of alleles contributing to the vancomycin tolerant phenotype. These findings allow a better understanding of evolution of antibiotic resistance and provide new information valuable for the epidemiological surveillance and control of *S. aureus* resistance in clinical environments. Most importantly, these data call into question the clinical reliability of *S. aureus* vancomycin susceptibility testing as it is currently performed in the clinical laboratory by providing a deeper understanding of why *S. aureus* resistance to vancomycin is rare in the laboratory yet vancomycin treatment failure is common in clinical practice.

## Methods

### Tolerization adaptive laboratory evolution (TALE)

TALE was performed as previously described^10^, with variations as noted. Four replicates of each starting strain (wild type and two media-adapted strains per media type) were inoculated from independent colonies on LB-agar plates. Cultures were grown in 15 ml working volume tubes which were heated to 37 °C and were aerobically stirred at 1100 rpm. Periodically, optical density readings at a 600-nanometer wavelength (OD_600_) were taken for each culture with a Tecan Sunrise reader plate, until the OD_600_ reached ~0.6 (approximately equivalent to an OD_600_ of 1 on a cm path length reader). At that time, 150 µl of the culture was passed to a fresh tube, to prevent the cells from reaching stationary phase. The passage volume was adjusted dynamically based on the actual OD_600_ at the time of passage, to keep the number of cells passed consistent. In addition, if the culture had grown for several consecutive flasks (~3 flasks), the vancomycin concentration in the next tube was increased. This stepwise increase began at 20% of the starting concentration but augmented over the course of the experiment. Growth rates were estimated for each tube by linear regression of the natural log of the optical density vs. time. Periodically throughout the experiment, culture aliquots were taken for long-term storage at −80 °C by mixing 800 µL of 50% glycerol with 800 µL of culture.

### Whole-genome sequencing

DNA sequencing was performed on clones and populations throughout the evolution, covering two or three timepoints of the evolution. Total genomic DNA was sampled from an overnight culture and extracted using a KingFisher Flex Purification system previously validated for the high throughput platform mentioned below^80^. Sequencing libraries were prepared using a miniaturized version of the Kapa HyperPlus Illumina-compatible library prep kit (Kapa Biosystems). DNA extracts were normalized to 5 ng total input per sample using an Echo 550 acoustic liquid handling robot (Labcyte Inc), and 1/10 scale enzymatic fragmentation, end-repair, and adapter-ligation reactions carried out using a Mosquito HTS liquid-handling robot (TTP Labtech Inc). Sequencing adapters were based on the iTru protocol^81^, in which short universal adapter stubs are ligated first and then sample-specific barcoded sequences added in a subsequent PCR step. Amplified and barcoded libraries were then quantified using a PicoGreen assay and pooled in approximately equimolar ratios before being sequenced on an Illumina HiSeq 4000 instrument.

The obtained sequencing reads were trimmed and filtered using AfterQC software, version 0.9.6^82^. Re-sequencing analysis for mutation identification was performed using the breseq bioinformatics pipeline^83^, version 0.31.1 and the *S. aureus* TCH1516 reference genome (GCA_000017085.1), reannotated using PATRIC^84^. ALEdb was used for mutation analysis^85^.

### Minimum inhibitory concentration

Strains were pre-cultured in the corresponding media (CA-MHB or RPMI + 10%LB) for ~5 h, and then inoculated to a final OD of 0.002 in media with or without vancomycin (Sigma). Growth was measured by following OD_600_ in a Bioscreen C Reader system with 150 μL per well. MIC_90_ was determined at 17 h post incubation. MIC_90_ is defined as the amount of drug required to inhibit ≥90% of the growth, therefore each drug-treated well was compared with untreated control well to determine the percentage of inhibition and identify the amount of drug causing ≥90% growth inhibition. The experiments were done in biological triplicates.

### Estimation of growth parameters

Growth parameters were estimated as previously described^86^. Briefly, lag phase was estimated by fitting the Baranyi growth model^87^ using nonlinear regression in R. A sensitivity analysis was run to exclude data points beyond a specific time threshold T, to avoid skewing the estimated parameters as a result of a possible cell death phase, secondary growth phase or noise. The sensitivity analysis ensured that the lag phase, exponential phase, and stationary phase only are taken into account in the fitting process, because all other growth/death phases are not explicitly modeled in Baranyi’s equation. Anova was run in R using aov() to test the null hypothesis that there is no difference in lag phase duration between pre-evolved strains (WT, STM2, STM3, STR1, and STR4) and vancomycin adapted strains.

### Resistance phenotype stability

Following TALE adaptation to vancomycin, a subset of the TALE strains (6 from each media) was further evolved in duplicate in the respective media for 21.79 ± 2.08 passages, 9.41 × 10^11^ ± 9.84 × 10^10^ CCDs. The final populations were then evaluated for their vancomycin susceptibility as described above.

### Transcriptomics

Eight TALE strains were selected for transcriptional analysis, along with their pre-evolved counterparts (wild type and one media-adapted strain per medium). Total RNA was sampled from biological duplicate cultures. The strains were grown in each respective media, with and without vancomycin (at 1/2 MIC). At OD 0.2, cultures without vancomycin were harvested. At the same OD, cultures were treated with 0.5× MIC of vancomycin, and harvested for RNA 30 min after. Harvesting of the cells consisted in mixing of 3 mL of culture with two volumes of Qiagen RNA-protect Bacteria Reagent (6 ml), vortexed for 5 s, incubated at room temperature for 5 min, and immediately centrifuged for 10 min at 17,500 r.p.m. The supernatant was decanted, and the cell pellet was stored at −80 °C. Total RNA was isolated using the Quick RNA Fungal/Bacterial Microprep (Zymo Research), following vendor procedures, including an on-column DNase treatment. RNA quality and purity were assessed using Nanodrop and Bioanalyzer RNA nano chip. Ribosomal RNA was removed from total RNA preparations using RNaseH. Then secondary structures in the ribosomal RNA were removed by heating to 90 degrees for 1 s, a set of 32-mer DNA oligo probes complementary to the 5S, 16S, and 23S subunits and spaced 9 bases apart were then annealed at 65 degrees followed by digestion with Hybridase (Lucigen), a thermostable RNAseH. The enzyme was added at 65 °C, the reaction incubated for 20 min at that temperature, then heated again to 90 °C for 1 s to remove remaining secondary structures, and returned to 65 °C for 10 min. The reaction was quickly quenched by the addition of guanidine thiocyanate while still at 65 °C before purifying the mRNA with a Zymo Research RNA Clean and Concentrator kit using their 200 nt cutoff protocol. Carryover oligos were removed with a DNAse I digestion which started at room temperature and gradually increased to 42 °C over a half hour. This was followed up with another column purification as stated above. The remaining RNA was used to build a cDNA library for sequencing using a KAPA Stranded RNA-seq Library Preparation Kit. The generated cDNA libraries were sent for Illumina sequencing on a HiSeq 4000.

The phred quality scores for the Illumina sequencing reads were generated using FastQC package^88^. Bowtie2 was used to align the raw reads to TCH1516 genome (GCA_000017085.1) and to calculate alignment percentage^89^. The aligned reads were then normalized to transcripts per million (TPM) with DESeq2^90^. The final expression values were log-transformed log_2_[TPM + 1] for visualization and analysis.

### Hemolysin production

Analysis of hemolysin production was performed by spotting 10 μL of a OD_600_ culture of 1 grown in CA-MHB onto 5% sheep blood agar plates. Plates were incubated at 37 °C for 24 h, followed by a 4 °C incubation for another 24 h. Hemolysin production was monitored after both incubations by observing the appearance of a clear halo.

### Surface charge

Quantification of the relative cell surface charge was performed using a cytochrome C (Sigma) binding assay, as previously described^91^. Briefly, cells were grown in CA-MHB until an OD_600_ of ~2, washed twice with MOPS buffer (20 mM, pH 7), and finally resuspended to an OD_600_ of 5. These were incubated for 10 min with 0.5 mg/ml (cytochrome C), which was subsequently removed by centrifugation. The amount of cytochrome C was spectrophotometrically quantified at 530 nm. The amount of cytochrome C bound to the cells can be used as a proxy for the cell surface charge.

### Autolysis assay

Evaluation of autolysis was performed using the Triton X-100-induced autolysis assay. Cells were grown to an OD_600_ of 1, washed twice with PBS buffer, and resuspended in PBS buffer containing 0.05 % Triton X-100. Cell suspensions were incubated at 37 °C and autolytic activity was measured by monitoring the OD_600_ every hour using Tecan Infinite 200 Pro microplate reader.

### Cell wall thickness

Cell wall thickness was measured using transmission electron microscopy (TEM). Cells were grown in CA-MHB at 37 °C overnight, diluted to an OD_600_ of 0.05, and allowed to grow until an OD_600_ of 0.5. Cell suspensions were harvested for 3 min at 10,000 r.p.m. at room temperature. Cell pellets were fixed with 2% glutaraldehyde in 0.10 M cacodylate buffer and further postfixed in 1% OsO_4_ in 0.1 M cacodylate buffer for 1 h on ice. The cells were stained all at once with 2% uranyl acetate for 1 h on ice, following which they were dehydrated in graded series of ethanol (50–100%) while remaining on ice. The cells were then subjected to 1 wash with 100% ethanol and 2 washes with acetone (10 min each) and embedded with Durcupan. Sections were cut at 60 nm on a Leica UCT ultramicrotome, and picked up on 300 mesh copper grids. Sections were post-stained with 2% uranyl acetate for 5 min and Sato’s lead stain for 1 min. EM images were taken on Jeol 1400 plus TEM with Gatan Digital camera 4kX4k camera. Image quantification was performed using ImageJ version 2.1.0/1.53c. Five intact cells were randomly selected for cell thickness measurements.

### Statistics and reproducibility

For statistical and reproducibility evaluation purposes, sample sizes were determined so that there would be 3 or more biological replicates for evolution experiments and according to the robotic capabilities per experiment, with a total of 12 different evolutions being performed per media type. For all other determinations, a minimum of triplicate samples were used in determination of biological measurements. Hyper mutators, i.e., lineages that have mutated genes within the mutagenesis repair systems, were excluded from biological determinations due to the possibility of higher-level impacts of such mutations. For minimal inhibitory concentration determinations experiments were performed in biological triplicates. Comparison of growth parameters was done using Anova to test the null hypothesis that there is no difference in lag phase duration between pre-evolved strains and vancomycin adapted strains. Evolution experiments to evaluate resistance phenotype stability and RNAseq experiments were performed in biological duplicates, while hemolysin production, surface charge, and autolysis were evaluated using biological triplicates. Statistical analysis of surface charge was performed using Student’s *t*-test.

### Reporting summary

Further information on research design is available in the Nature Research Reporting Summary linked to this article.

## Supplementary information

Supplementary Information

Supplementary Data 1

Supplementary Data 2

Supplementary Data 3

Supplementary Data 4

Supplementary Data 5

Supplementary Data 6

Supplementary Data 7

Supplementary Data 8

Supplementary Data 9

Supplementary Data 10

Description of Additional Supplementary Files

Reporting Summary

## Data Availability

Newly determined DNA sequence data were deposited in the NCBI database under BioProject PRJNA521551, accession numbers SRR8552163 to SRR8552250. All RNA-seq data have been deposited to the GEO database (record GSE149213) and Short Read Archive (SRA), RNA-seq data accession numbers SRX8164260 to SRX8164307. All other data are available from the corresponding author on reasonable request.

## References

[1] Organization, W. H. Antimicrobial resistance: global report on surveillance. *Who* 8 (2014).

[2] Centers for Disease Control and Prevention (U.S.). Antibiotic resistance threats in the United States, 2019. 10.15620/cdc:82532 (2019).

[3] Alcock BP (2020). CARD 2020: antibiotic resistome surveillance with the comprehensive antibiotic resistance database. Nucleic Acids Res..

[4] Pollack LA, Srinivasan A (2014). Core elements of hospital antibiotic stewardship programs from the Centers for Disease Control and Prevention. Clin. Infect. Dis..

[5] Martens, E. & Demain, A. L. The antibiotic resistance crisis, with a focus on the United States. *J. Antibiot*. **70**, 520–526 (2017). 10.1038/ja.2017.30 28246379

[6] Mitsakakis K, Kaman WE, Elshout G, Specht M, Hays JP (2018). Challenges in identifying antibiotic resistance targets for point-of-care diagnostics in general practice. Future Microbiol..

[7] Farha MA, French S, Stokes JM, Brown ED (2018). Bicarbonate alters bacterial susceptibility to antibiotics by targeting the proton motive force. ACS Infect. Dis..

[8] Ersoy SC (2017). Correcting a fundamental flaw in the paradigm for antimicrobial susceptibility testing. EBioMedicine.

[9] Kumaraswamy M (2016). Standard susceptibility testing overlooks potent azithromycin activity and cationic peptide synergy against MDR *Stenotrophomonas maltophilia*. J. Antimicrob. Chemother..

[10] Mohamed ET (2017). Generation of a platform strain for ionic liquid tolerance using adaptive laboratory evolution. Microb. Cell Fact..

[11] Dragosits M, Mattanovich D (2013). Adaptive laboratory evolution—principles and applications for biotechnology. Microb. Cell Fact..

[12] Salazar, M. J. et al. Genetic determinants enabling medium-dependent adaptation to nafcillin in methicillin-resistant *Staphylococcus aureus*. *mSystems* **5**, e00828-19 (2020). 10.1128/mSystems.00828-19 PMC7112963 32234776

[13] Mwangi MM (2007). Tracking the in vivo evolution of multidrug resistance in *Staphylococcus aureus* by whole-genome sequencing. Proc. Natl Acad. Sci. USA.

[14] Pader, V. et al. *Staphylococcus aureus* inactivates daptomycin by releasing membrane phospholipids. *Nat. Microbiol.* **2**, 16194 (2016). 10.1038/nmicrobiol.2016.194 27775684

[15] Howden BP, Davies JK, Johnson PDR, Stinear TP, Grayson ML (2010). Reduced vancomycin susceptibility in *Staphylococcus aureus*, including vancomycin-intermediate and heterogeneous vancomycin-intermediate strains: resistance mechanisms, laboratory detection, and clinical implications. Clin. Microbiol. Rev..

[16] Sorrell TC, Packham DR, Shanker S, Foldes M, Munro R (1982). Vancomycin therapy for methicillin-resistant *Staphylococcus aureus*. Ann. Intern. Med..

[17] McGuinness WA, Malachowa N, DeLeo FR (2017). Vancomycin resistance in *Staphylococcus aureus*. Yale J. Biol. Med..

[18] Hidayat LK, Hsu DI, Quist R, Shriner KA, Wong-Beringer A (2006). High-dose vancomycin therapy for methicillin-resistant *Staphylococcus aureus* infections: efficacy and toxicity. Arch. Intern. Med..

[19] Hiramatsu K (1997). Dissemination in Japanese hospitals of strains of *Staphylococcus aureus* heterogeneously resistant to vancomycin. Lancet.

[20] Howe RA, Bowker KE, Walsh TR, Feest TG, MacGowan AP (1998). Vancomycin-resistant *Staphylococcus aureus*. Lancet.

[21] Gonzalez BE (2005). Severe staphylococcal sepsis in adolescents in the era of community-acquired methicillin-resistant *Staphylococcus aureus*. Pediatrics.

[22] McKee TJ, Komarova SV (2017). Is it time to reinvent basic cell culture medium?. Am. J. Physiol. Cell Physiol..

[23] Ishii K (2015). Phenotypic and genomic comparisons of highly vancomycin-resistant *Staphylococcus aureus* strains developed from multiple clinical MRSA strains by in vitro mutagenesis. Sci. Rep..

[24] Gardete, S. et al. Genetic pathway in acquisition and loss of vancomycin resistance in a methicillin resistant *Staphylococcus aureus* (MRSA) strain of clonal type USA300. *PLoS Pathog*. **8**, e1002505 (2012). 10.1371/journal.ppat.1002505 PMC3271070 22319446

[25] Hershberg, R. Mutation—the engine of evolution: studying mutation and its role in the evolution of bacteria. *Cold Spring Harb. Perspect.**Biol.* **7***,* a018077 (2015). 10.1101/cshperspect.a018077 PMC4563715 26330518

[26] Hershberg R, Petrov DA (2010). Evidence that mutation is universally biased towards AT in bacteria. PLoS Genet..

[27] Hildebrand F, Meyer A, Eyre-Walker A (2010). Evidence of selection upon genomic GC-content in bacteria. PLoS Genet..

[28] Ban C, Yang W (1998). Crystal structure and ATPase activity of MutL: implications for DNA repair and mutagenesis. Cell.

[29] Glickman BW, Radman M (1980). *Escherichia coli* mutator mutants deficient in methylation-instructed DNA mismatch correction. Proc. Natl Acad. Sci. USA.

[30] Hu, Q., Peng, H. & Rao, X. Molecular events for promotion of vancomycin resistance in vancomycin intermediate *Staphylococcus aureus*. *Front. Microbiol*. **7**, 1601 (2016). 10.3389/fmicb.2016.01601 PMC5062060 27790199

[31] Kato Y, Suzuki T, Ida T, Maebashi K (2010). Genetic changes associated with glycopeptide resistance in *Staphylococcus aureus*: predominance of amino acid substitutions in YvqF/VraSR. J. Antimicrob. Chemother..

[32] Cameron DR (2012). Serine/threonine phosphatase Stp1 contributes to reduced susceptibility to vancomycin and virulence in *Staphylococcus aureus*. J. Infect. Dis..

[33] Koch G (2014). Evolution of resistance to a last-resort antibiotic in *Staphylococcus aureus* via bacterial competition. Cell.

[34] Howden BP (2011). Evolution of multidrug resistance during *Staphylococcus aureus* infection involves mutation of the essential two component regulator WalKR. PLoS Pathog..

[35] Vidaillac C (2013). Alternative mutational pathways to intermediate resistance to vancomycin in methicillin-resistant *Staphylococcus aureus*. J. Infect. Dis..

[36] Hu J, Zhang X, Liu X, Chen C, Sun B (2015). Mechanism of reduced vancomycin susceptibility conferred by *walK* mutation in community-acquired methicillin-resistant *Staphylococcus aureus* strain MW2. Antimicrob. Agents Chemother..

[37] Peng, H. et al. WalK(S221P), a naturally occurring mutation, confers vancomycin resistance in VISA strain XN108. *J. Antimicrobial Chemother.* dkw518 10.1093/jac/dkw518 (2016). 10.1093/jac/dkw518 27999059

[38] Kuroda M (2003). Two-component system VraSR positively modulates the regulation of cell-wall biosynthesis pathway in *Staphylococcus aureus*. Mol. Microbiol..

[39] Hiramatsu K (2001). Vancomycin-resistant *Staphylococcus aureus*: a new model of antibiotic resistance. Lancet Infect. Dis..

[40] Wang QM (2001). Identification and characterization of a monofunctional glycosyltransferase from *Staphylococcus aureus*. J. Bacteriol..

[41] Jousselin A (2015). The *Staphylococcus aureus* chaperone PrsA is a new auxiliary factor of oxacillin resistance affecting penicillin-binding protein 2A. Antimicrob. Agents Chemother..

[42] Villanueva M (2018). Sensory deprivation in *Staphylococcus aureus*. Nat. Commun..

[43] Łęski TA, Tomasz A (2005). Role of penicillin-binding Protein 2 (PBP2) in the antibiotic susceptibility and cell wall cross-linking of *Staphylococcus aureus*: evidence for the cooperative functioning of PBP2, PBP4, and PBP2A. J. Bacteriol..

[44] Oku Y, Kurokawa K, Ichihashi N, Sekimizu K (2004). Characterization of the *Staphylococcus aureus mprF* gene, involved in lysinylation of phosphatidylglycerol. Microbiology.

[45] Chen F-J (2018). Effect of a point mutation in *mprF* on susceptibility to daptomycin, vancomycin, and oxacillin in an MRSA clinical strain. Front. Microbiol..

[46] Goffin C, Ghuysen JM (1998). Multimodular penicillin-binding proteins: an enigmatic family of orthologs and paralogs. Microbiol. Mol. Biol. Rev..

[47] Murakami K (1994). Nucleotide sequence of the structural gene for the penicillin-binding protein 2 of *Staphylococcus aureus* and the presence of a homologous gene in other staphylococci. FEMS Microbiol. Lett..

[48] Sieradzki K, Tomasz A (1999). Gradual alterations in cell wall structure and metabolism in vancomycin-resistant mutants of *Staphylococcus aureus*. J. Bacteriol..

[49] Xia G, Wolz C (2014). Phages of *Staphylococcus aureus* and their impact on host evolution. Infect. Genet. Evol..

[50] Goerke C (2009). Diversity of prophages in dominant *Staphylococcus aureus* clonal lineages. J. Bacteriol..

[51] Verkaik NJ (2011). Immune evasion cluster-positive bacteriophages are highly prevalent among human *Staphylococcus aureus* strains, but they are not essential in the first stages of nasal colonization. Clin. Microbiol. Infect..

[52] van Wamel WJB, Rooijakkers SHM, Ruyken M, van Kessel KPM, van Strijp JAG (2006). The innate immune modulators staphylococcal complement inhibitor and chemotaxis inhibitory protein of *Staphylococcus aureus* are located on -hemolysin-converting bacteriophages. J. Bacteriol..

[53] Read TD (2018). USA300 *Staphylococcus aureus* persists on multiple body sites following an infection. BMC Microbiol..

[54] Tran, P. M., Feiss, M., Kinney, K. J. & Salgado-Pabón, W. ϕSa3mw prophage as a molecular regulatory switch of *Staphylococcus aureus* β-toxin production. *J. Bacteriol*. **201**, e00766-18 (2019). 10.1128/JB.00766-18 PMC6597384 30962356

[55] Boyle-Vavra S, Yin S, Jo DS, Montgomery CP, Daum RS (2013). VraT/YvqF is required for methicillin resistance and activation of the VraSR regulon in *Staphylococcus aureus*. Antimicrob. Agents Chemother..

[56] Cameron DR, Jiang JH, Kostoulias X, Foxwell DJ, Peleg AY (2016). Vancomycin susceptibility in methicillin-resistant *Staphylococcus aureus* is mediated by YycHI activation of the WalRK essential two-component regulatory system. Sci. Rep..

[57] Delauné A (2012). The WalKR system controls major staphylococcal virulence genes and is involved in triggering the host inflammatory response. Infect. Immun..

[58] Poudel S (2020). Revealing 29 sets of independently modulated genes in *Staphylococcus aureus*, their regulators, and role in key physiological response. Proc. Natl Acad. Sci. USA.

[59] Balibar CJ (2010). *cwrA*, a gene that specifically responds to cell wall damage in *Staphylococcus aureus*. Microbiology.

[60] McAleese F (2006). Overexpression of genes of the cell wall stimulon in clinical isolates of *Staphylococcus aureus* exhibiting vancomycin-intermediate- *S. aureus*-type resistance to vancomycin. J. Bacteriol..

[61] Ernst, C. M. et al. Gain-of-function mutations in the phospholipid flippase MprF confer specific daptomycin resistance. *mBio* **9**, e01659-18 (2018). 10.1128/mBio.01659-18 PMC6299216 30563904

[62] Ernst, C. M. & Peschel, A. MprF-mediated daptomycin resistance. *Int. J. Med. Microbiol*. 10.1016/j.ijmm.2019.05.010 (2019). 10.1016/j.ijmm.2019.05.010 31182276

[63] Fey PD (2013). A genetic resource for rapid and comprehensive phenotype screening of nonessential *Staphylococcus aureus* genes. MBio.

[64] Podolsky SH (2018). The evolving response to antibiotic resistance (1945–2018). Palgrave Commun..

[65] Howden BP (2008). Different bacterial gene expression patterns and attenuated host immune responses are associated with the evolution of low-level vancomycin resistance during persistent methicillin-resistant *Staphylococcus aureus* bacteraemia. BMC Microbiol..

[66] McAleese F (2006). Overexpression of genes of the cell wall stimulon in clinical isolates of *Staphylococcus aureus* exhibiting vancomycin-intermediate-S. aureus-type resistance to vancomycin. J. Bacteriol..

[67] Sakoulas G (2002). Accessory gene regulator (*agr*) locus in geographically diverse *Staphylococcus aureus* isolates with reduced susceptibility to vancomycin. Antimicrob. Agents Chemother..

[68] McCallum N, Stutzmann Meier P, Heusser R, Berger-Bächi B (2011). Mutational analyses of open reading frames within the *vraSR* operon and their roles in the cell wall stress response of *Staphylococcus aureus*. Antimicrob. Agents Chemother..

[69] Dubrac S, Boneca IG, Poupel O, Msadek T (2007). New insights into the WalK/WalR (YycG/YycF) essential signal transduction pathway reveal a major role in controlling cell wall metabolism and biofilm formation in *Staphylococcus aureus*. J. Bacteriol..

[70] Strahl H, Hamoen LW (2010). Membrane potential is important for bacterial cell division. Proc. Natl Acad. Sci. USA.

[71] Li B, Qiu Y, Shi H, Yin H (2016). The importance of lag time extension in determining bacterial resistance to antibiotics. Analyst.

[72] Lin L (2015). Azithromycin synergizes with cationic antimicrobial peptides to exert bactericidal and therapeutic activity against highly multidrug-resistant Gram-negative bacterial pathogens. EBioMedicine.

[73] Levine DP (2006). Vancomycin: a history. Clin. Infect. Dis..

[74] Giuliano C, Haase KK, Hall R (2010). Use of vancomycin pharmacokinetic–pharmacodynamic properties in the treatment of MRSA infections. Expert Rev. Anti. Infect. Ther..

[75] Sabat AJ (2018). Daptomycin resistant *Staphylococcus aureus* clinical strain with novel non-synonymous mutations in the *mprF* and *vraS* genes: a new insight into daptomycin resistance. Front. Microbiol..

[76] Costa SS (2018). Genetic diversity of *norA*, coding for a main efflux pump of *Staphylococcus aureus*. Front. Genet..

[77] Shore AC (2010). Identification and characterization of the multidrug resistance gene cfr in a panton-valentine leukocidin-positive sequence type 8 methicillin-resistant *Staphylococcus aureus* IVa (USA300) isolate. Antimicrobial Agents Chemother..

[78] Machado, H. et al. A defined minimal medium for systems analyses of *Staphylococcus aureus* reveals strain-specific metabolic requirements. *Appl. Environ. Microbiol*. **85**, e01773-19 (2019). 10.1128/AEM.01773-19 PMC6803309 31471305

[79] McAdam PR, Holmes A, Templeton KE, Fitzgerald JR (2011). Adaptive evolution of *Staphylococcus aureus* during chronic endobronchial infection of a cystic fibrosis patient. PLoS ONE.

[80] Marotz C (2017). DNA extraction for streamlined metagenomics of diverse environmental samples. Biotechniques.

[81] Glenn TC (2019). Adapterama I: universal stubs and primers for 384 unique dual-indexed or 147,456 combinatorially-indexed Illumina libraries (iTru & iNext). PeerJ.

[82] Chen S (2017). AfterQC: automatic filtering, trimming, error removing and quality control for fastq data. BMC Bioinforma..

[83] Deatherage DE, Barrick JE (2014). Identification of mutations in laboratory-evolved microbes from next-generation sequencing data using breseq. Methods Mol. Biol..

[84] Brettin T (2015). RASTtk: a modular and extensible implementation of the RAST algorithm for building custom annotation pipelines and annotating batches of genomes. Sci. Rep..

[85] Phaneuf, P. V., Gosting, D., Palsson, B. O. & Feist, A. M. ALEdb 1.0: a database of mutations from adaptive laboratory evolution experimentation. *Nucleic Acids Res*. **47**, D1164–D1171 (2018). 10.1093/nar/gky983 PMC6323966 30357390

[86] Anand A (2020). OxyR is a convergent target for mutations acquired during adaptation to oxidative stress-prone metabolic states. Mol. Biol. Evol..

[87] Baranyi J, Roberts TA (1994). A dynamic approach to predicting bacterial growth in food. Int. J. Food Microbiol..

[88] Andrews, S. & Others. *FastQC: a Quality Control Tool for High Throughput Sequence Data* (2010).

[89] Langmead B, Salzberg SL (2012). Fast gapped-read alignment with Bowtie 2. Nat. Methods.

[90] Love MI, Huber W, Anders S (2014). Moderated estimation of fold change and dispersion for RNA-seq data with DESeq. 2. Genome Biol..

[91] Peschel A (1999). Inactivation of the *dlt* operon in *Staphylococcus aureus* confers sensitivity to defensins, protegrins, and other antimicrobial peptides. J. Biol. Chem..

