# Supplementary material for: Environmental conditions dictate differential evolution of vancomycin resistance in Staphylococcus aureus
Source: Commun Biol. 2021 Jun 25;4:793. doi: 10.1038/s42003-021-02339-z (PMC8233327; doi:10.1038/s42003-021-02339-z)

**Supplementary Table 1 -** Minimum inhibitory concentration (MIC_90_) of *S. aureus* starting strains and TALE-derived strains in the same environmental conditions used for tolerance evolution, CA-MHB or RPMI+. Post-TALE refers to the MIC_90_of the population after 21.79 ± 2.08 passages (9.41x10^11^ ± 9.84x10^10^ CCDs) in the media used for evolution, without vancomycin. The post-TALE evolution was performed in duplicate for each of the end-point clones.

|  | **MIC_90_  (μg/mL) in CA-MHB** | |  |  | **MIC_90_  (μg/mL) in RPMI+** | |
| --- | --- | --- | --- | --- | --- | --- |
| **Strain** | **Starting strain / TALE** | **post-TALE** |  | **Strain** | **Starting strain / TALE** | **post-TALE** |
| **WT** | 1 | - |  | **WT** | 2 | - |
| **SVAM_A2** | 4 | ND |  | **SVAR_A1** | 4 | ND |
| **SVAM_A3** | 8 | 4 / 4 |  | **SVAR_A2** | 8-16 | 8 / 8 |
| **STM2** | 1 | - |  | **SVAR_A3** | 16 | 16 / 16 |
| **SVAM_A6** | 4-8 | 2-4 / 4 |  | **SVAR_A10** | 8 | ND |
| **SVAM_A7** | 8 | 4 / 4 |  | **STR1** | 2 | - |
| **SVAM_A8** | 4 | ND |  | **SVAR_A4** | 8-16 | 8 / 8 |
| **STM3** | 1 | - |  | **SVAR_A5** | 8-16 | 8-16 / 8 |
| **SVAM_A9** | 8 | 4 / 4 |  | **SVAR_A11** | 8 | ND |
| **SVAM_A10** | 8 | 2 / 2 |  | **STR4** | 4 | - |
| **SVAM_A11** | 4 | ND |  | **SVAR_A7** | 8-16 | 8 -16 / 16 |
| **SVAM_A12** | 8 | 4 / 4 |  | **SVAR_A8** | 8 | ND |
|  |  |  |  | **SVAR_A12** | 8 | 8 / 8 |

**Supplementary Figure 1 –** Characteristics of vancomycin TALE strains. (a) An image of a plate displaying hemolytic activity after 24 h incubation at 37 °C for starting strains and vancomycin TALE strains (b) Autolysis of whole cells of *S. aureus*. Mid-exponential-phase cultures were resuspended in 0.05 M Tris-HCl (pH 7.2) containing 0.05% Triton X-100 and were incubated at 30°C. Absorbance was measured every hour and percentage to initial absorbance calculated. (c) Transmission electron microscopy (TEM) pictures of strains in exponential growth phase. (d) Measurement of cell wall thickness of several strains using representative TEM pictures, statistical analysis provided as Supplementary Table 2.

**Supplementary Table 2 –** Statistical analysis of the cell wall thickness measurements for the duration of vancomycin TALE strains, using a parametric unpaired t-test with Welch’s correction.

| **Sample pair** | **p-value** | **p-value summary** |
| --- | --- | --- |
| STR1 vs WT | 0.1406 | ns |
| SVAM_A3 vs WT | 0.0167 | * |
| SVAM_A12 vs WT | <0.0001 | **** |
| SVAR_A3 vs WT | <0.0001 | **** |
| SVAR_A5 vs WT | 0.0788 | ns |
| SVAR_A3 vs STR1 | <0.0001 | **** |
| SVAR_A5 vs STR1 | 0.6685 | ns |
| SVAR_A5 vs SVAR_A3 | <0.0001 | **** |

**Supplementary Figure 2 –** Growth profiling of evolution starting strains (WT and STM2) and vancomycin adapted strains (SVAM_A2) in the absence and presence of vancomycin (0.5 µg/mL). An example of the increase in lag phase.

**Supplementary Table 3 –** ANOVA statistical analysis of the lag-phase duration of starting strains and evolved strains in the same media as that used for tolerization.

| **vancomycin.ug.ml** | **Starting_strain** | **F.value** | **Pr..F.** |
| --- | --- | --- | --- |
| 0 | STM2 | 4.37324747923248 | 0.062998644983095 |
| 0 | STM3 | 8.21404843584888 | 0.0132439301947123 |
| 0 | STR1 | 55.9776079042055 | 2.10636199647478E-05 |
| 0 | STR4 | 18.2400821654054 | 0.00163482854999148 |
| 0.5 | STM2 | 180.845045483513 | 9.94033084285709E-08 |
| 0.5 | STM3 | 163.726248981813 | 9.6409038322023E-09 |
| 0.5 | STR1 | 355.069596153815 | 3.83918455769995E-09 |
| 0.5 | STR4 | 198.53651002671 | 6.36874197639287E-08 |
| 0 | WT(MHB) | 533.791403846887 | 7.21720540302604E-08 |
| 0 | WT(RPMI) | 11.7094080873006 | 0.00652657253351374 |
| 0.5 | WT(MHB) | 499.257469737133 | 9.09544723581264E-08 |
| 0.5 | WT(RPMI) | 5.55414886661091 | 0.0401771670140192 |

**Supplementary Figure 3 –** Venn diagram of the key mutated genes (≥ 2 instances) in the two utilized media conditions (i.e., CA-MHB and RPMI+).


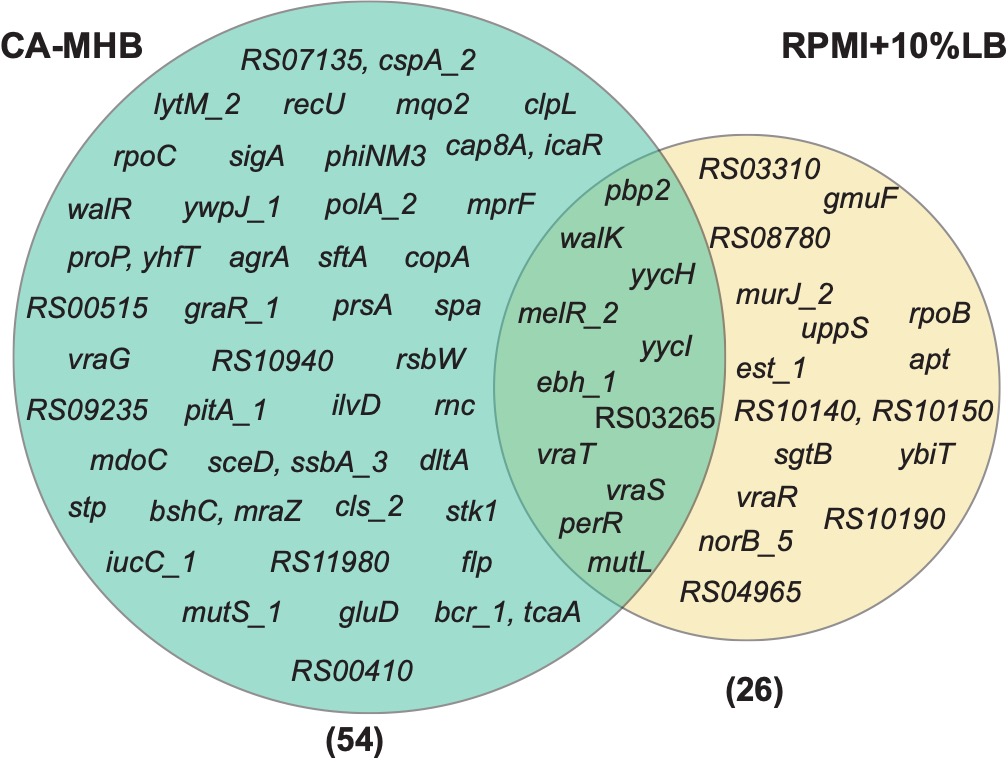

Supplement: Supplementary file 1 — Supplementary Information [file 42003_2021_2339_MOESM1_ESM.docx]
