# Supplementary material for: Environmental conditions dictate differential evolution of vancomycin resistance in Staphylococcus aureus
Source: Commun Biol. 2021 Jun 25;4:793. doi: 10.1038/s42003-021-02339-z (PMC8233327; doi:10.1038/s42003-021-02339-z)
Supplement: Supplementary file 12 — Description of Additional Supplementary Files [file 42003_2021_2339_MOESM12_ESM.pdf]

Title: Supplementary Data 1
Description: Mutations identified for TALE-derived strains after tolerization to vancomycin in CA-MHB. Nomenclature example A1 F29 I1 R1 = ALE 1 Flask 29 Isolate (I1=clone, I0=population) Replicate 1.

Title: Supplementary Data 2
Description: Mutations identified for TALE-derived strains after tolerization to vancomycin in RPMI+. Nomenclature example A1 F29 I1 R1 = ALE 1 Flask 29 Isolate (I1=clone, I0=population) Replicate 1.

Title: Supplementary Data 3
Description: Trajectories of Tolerization Adaptive Laboratory Evolution of SVAM strains

Title: Supplementary Data 4
Description: Trajectories of Tolerization Adaptive Laboratory Evolution of SVAR strains

Title: Supplementary Data 5
Description: Growth parameters calculated for TALE strains

Title: Supplementary Data 6
Description: Log_2 vancomycin MIC values of evolved strains from all TALE conditions tested in both media types

Title: Supplementary Data 7
Description: Differentially Expressed genes identified in the RNAseq dataset

Title: Supplementary Data 8
Description: Quantification of cytochrome C bound to the cells

Title: Supplementary Data 9
Description: Autolysis measured for of SVAM strains

Title: Supplementary Data 10
Description: Autolysis measured for of SVAR strains
